# Sex and Gender Health Educational Tenets: A Report from the 2020 Sex and Gender Health Education Summit

**DOI:** 10.1089/jwh.2022.0222

**Published:** 2022-07-12

**Authors:** Juliana M. Kling, Rebecca Sleeper, Eliza Lo Chin, Mary K. Rojek, Alyson J. McGregor, Lorie Richards, Ann Bradley Mitchell, Christina Stasiuk, Kimberly Templeton, Joanne Prasad, Sandra Pfister, Connie B. Newman

**Affiliations:** ^1^ Division of Women's Health Internal Medicine, Department of Medicine, Mayo Clinic Arizona, Scottsdale Arizona, USA.; ^2^ Jerry H. Hodge School of Pharmacy, Texas Tech University Health Sciences Center, Lubbock, Texas, USA.; ^3^ American Medical Women's Association, Schaumburg, Illinois, USA.; ^4^ Division of Sex and Gender, Department of Emergency Medicine, The Warren Alpert Medical School of Brown University, Providence, Rhode Island, USA.; ^5^ Department of Occupational and Recreational Therapies, College of Health, University of Utah, Salt Lake City, Utah, USA.; ^6^ Thomas Jefferson University–College of Nursing, Jefferson University, Philadelphia, Pennsylvania, USA.; ^7^ Cigna, Senior Medical Director, Clinical Performance and Quality, Philadelphia, Pennsylvania, USA.; ^8^ Department of Oral and Craniofacial Sciences, Department of Orthopedic Surgery, University of Kansas Medical Center, Kansas City, Kansas, USA.; ^9^ Department of Oral and Craniofacial Sciences, School of Dental Medicine, University of Pittsburgh, Pittsburgh, Pennsylvania, USA.; ^10^ Department of Pharmacology and Toxicology, The Kern Institute for the Transformation of Medical Education, Medical College of Wisconsin, Milwaukee, Wisconsin, USA.; ^11^ Department of Medicine, NYU Grossman School of Medicine, New York, New York, USA.

**Keywords:** sex and gender, health education, interprofessional

## Abstract

**Background:**

Sex as a biological variable and gender as a sociocultural variable influence many health conditions and outcomes. However, they have not been incorporated systematically into education across health professions.

**Methods:**

Areas of knowledge and abilities that apply to sex and gender education across health professions were summarized from the 2015 and 2018 Sex and Gender Health Education Summits.

**Results:**

Using this summary, draft tenets were developed by facilitated interprofessional discussion groups at the 2020 Summit, and then reviewed, edited, and refined by a writing group who recommended four tenets that health care professionals should be able to do: (1) demonstrate knowledge of sex and gender specific health (SGSH), (2) evaluate literature and the conduct of research for incorporation of sex and gender, (3) incorporate sex and gender considerations into clinical decision making, and (4) demonstrate patient advocacy with respect to sex and gender.

**Conclusion:**

These tenets provide the framework for collaborative interprofessional education about SGSH. Individual professions can also use the tenets to develop practice-specific competencies, competency statements, and/or assessment benchmarks within the structures of their respective accrediting bodies to advance the health of women, men, and sex and gender minority persons. Interprofessional collaborations are key for sharing best practices in development, curricular integration, and dissemination.

## Introduction

Health care practice has historically been based on research conducted on males, and health profession education has been taught from that perspective. In 1990, the United States National Institutes of Health (NIH) established the Office of Research on Women's Health (ORWH) to address this deficit. The NIH Revitalization Act of 1993 was passed to ensure the adequate representation of women in NIH-funded research.^1^ These actions ultimately led to requirements for inclusion of both males/men and females/women in all phases of preclinical and clinical federally funded studies and analysis of data by sex.

A foundational 2001 report from the Institute of Medicine reinforced the position that sex differences affect human health throughout the lifespan and should be a research priority.^2^ In 2015, a new NIH policy recommended that researchers should consider sex as a biological variable (SABV) in basic science studies.^3^ Nevertheless, most preclinical and clinical research studies still do not adequately represent females/women, disaggregate, and analyze and report data by sex, or account for gender influences.^4^ This lack is due, in part, to funding concerns, deficiency of knowledge or recognition of the importance of evaluating these differences,^5^ or potentially other factors such as lack of support for integration, and sexism and genderism.

Together, both sex and gender impact health outcomes for all persons because every cell has a sex, and every person has a gender. Specifically, they have an impact on anatomy, physiology, pathophysiology, the clinical presentation of disease, access to treatment, and the efficacy and safety of treatments. The term “sex” refers to biological characteristics such as sex chromosomes and their expression, reproductive organs (ovaries, testes), and endogenous hormones, which may vary in concentrations and function. These characteristics categorize humans as female, male, intersex, and hermaphrodite.^6^ Gender is a sociocultural variable and refers to socially constructed roles, behaviors, expressions, and identities.^7^ It influences individuals' behaviors, risk factors, perceptions of disease (by patients and health care professionals), and willingness and ability to seek health care.^8^

Gender norms can vary across time and subcultures and can also have an impact on health through epigenetic processes.^9^ Gender identity and gender expression are nonbinary and can shift over time in individuals.^10^ Efforts to measure gender are underway to guide health care and health policy.^11^ While both sex and gender have an impact on health for all persons, they have an additional impact on access to and quality of care for members of sexual and gender minority persons due to biases related to sexuality and gender.

To date, evidence of sex and gender differences, along with the basic concepts of sex and gender, has not been routinely integrated into teaching and training in health professions in the United States, and thus do not yet fully inform clinical practice.^12^ Furthermore, sex and gender concepts are just beginning to be explored within interprofessional education.^13^ Efforts to do so began in 2018 at an international summit and continued beyond, ultimately leading to the development of educational tenets aimed at providing a unified message for broad interprofessional education. This article describes how an interprofessional team of sex and gender health education experts developed these tenets for use by all health care professions for the creation of sex and gender health interprofessional curricula.

## Approach: Rationale for Integration of Sex and Gender Health Into the Curriculum

An initial workshop held at the Mayo Clinic in 2012 brought together 13 groups, representing institutions, organizations, and government agencies from the United States and Canada, around the common goal of facilitating the integration of sex- and gender-based content into medical education and training. In 2015, the first large-scale national effort to advance sex- and gender-based medical education was convened at the Sex and Gender Medical Education Summit held at the Mayo Clinic. A partnership between the American Medical Women's Association (AMWA), the Laura W. Bush Institute for Women's Health (LWBIWH) at the Texas Tech University Health Sciences Center, the Mayo Clinic, and the Society for Women's Health Research enabled this summit. One hundred forty-eight in-person attendees and 27 webcast attendees representing 99 U.S. institutions, 12 international schools, and 15 professional organizations, as well as student and nonprofit organizations and government agencies gathered to create a roadmap for integrating sex and gender content into medical education.

At that Summit, Ann Bonham, PhD, then Chief Scientific Officer at the Association of American Medical Colleges, stated “Recognizing that sex matters in biological processes in health and disease is about good science and providing high quality care to both women and men.”^14^ Marjorie Jenkins, MD, MEdHP, then Chief Scientific Officer of the LWBIWH at Texas Tech University Health Sciences Center and Summit Co-Chair reminded attendees, “There is no research discovery, no matter how amazing, that will save a patient's life unless it first traverses a learning environment. We must assist current health professionals in recognizing the increasing body of knowledge around sex and gender differences, and even more so, passing knowledge into medical education.”^14^ Summit attendees discussed strategies for faculty and leaders to use when advocating for improved medical education within their home institutions.

In 2018, the Sex and Gender Health Education Summit was held at the University of Utah. The 2018 Summit represented an expansion of the 2015 Summit to an interprofessional collaboration of educators and thought leaders of multiple profession, including dentistry, medicine, nursing, occupational therapy, pharmacy, and physical medicine. This summit was a partnership between AMWA, LWBIWH, the Mayo Clinic, and the University of Utah. Two hundred forty-six in-person attendees from 144 U.S. health sciences schools and health care centers, 8 international schools, 6 associations and organizations, and 3 government agencies gathered to develop strategies for assessing and integrating sex and gender content into curricula.

Summit attendees also discussed comprehensive strategies for leading and sustaining curricular change. Leslie Halpern, DDS, MD, PhD, MPH, noted the significance of interprofessional education and collaborative practice initiatives stating that “Sex and gender is such an important part of pedagogical training because it is health determinants that weave within the tapestry of interprofessional education.”^15^ This first interprofessional summit on sex and gender enabled exchanges from various health professions through lectures and workshops composed of members from various professions.

Integrating sex and gender concepts into training programs across all health professions is critical to achieving universal adoption of sex and gender specific health (SGSH) into clinical practice. Knowledge of sex and gender concepts is essential in any scientific field that advances knowledge of pathophysiology, disease prevention, disease management, and health behaviors, and in understanding overall health. The importance of adopting sex and gender health differences into curricula has been previously discussed.^12–16^ Several examples of profession-specific institutional initiatives to define competencies or learning outcomes related to sex and gender have also been reported in the medical literature.^17,18^ Apart from these efforts, limited work has been done to integrate gender and sex within interprofessional educational activities.^19,20^

At the 2018 summit, the task was to incorporate Sex and Gender Health Education (SGHE) into interprofessional activities, but as much as we may have delivered an educational program to the summit audience, we received an education ourselves. Creating interprofessional experiences in SGHE does not happen when we do not speak the same language, and when our accreditation standards do not speak the same language. There is utility in each profession individually applying the tenets in their own respective teaching, but it also extends to the incorporation of SGHE in Interprofessional Education (IPE) as well.

### Pathway to Development of Tenets for Sex and Gender Specific Health Education

The 2015 and 2018 Summits were foundational in incorporating SGSH into health professions curricula. As shown at the 2018 Summit, sex and gender fit into multiple competency domains in all health professions. However, there has not been uniform progress in curricular reform, nor establishment of a consensus definition of SGSH education that applies to all health professions learners. Obstacles to health care-wide acceptance of SGSH education and training may include a lack of understanding of the principles of SGSH education, the absence of consistent educational competencies, a lack of profession-specific accreditation standards, and a lack of awareness by curriculum leaders, faculty, and researchers of the importance and impact of this topic, and difficulties in implementing curricular change.^21^

To overcome these challenges, a critical objective of the 2020 Sex and Gender Health Education Summit was to arrive at a consensus about guiding principles that would create a framework for achieving universal inclusion of sex and gender content in the teaching and training of future health care professionals across all disciplines. The 2020 Summit was co-sponsored by AMWA, LWBIWH, Mayo Clinic, and Thomas Jefferson University. It was held virtually due to the COVID-19 pandemic. Total registration was comparable to the 2018 Summit (2018 Summit was 246, and 2020 was 245). The key question posed at the 2020 Summit was “What is it that all health professionals should know and do with respect to sex and gender specific health?” It was agreed that defining principles or tenets that are universal to all health professions would allow a systematic approach for incorporating sex and gender based content into the curricula across all health professions. This strategy was modeled after the work of the Interprofessional Education Collaborative (IPEC),^22^ which had evaluated individual efforts and curricular content in relation to interprofessional collaboration from a variety of health professions. The IPEC defined and subsequently updated, in 2011 and 2016, respectively, four domains for collaborative practice: values and ethics, roles and responsibilities, interprofessional communication, and teams and teamwork.^22^ The four domains from IPEC allowed for the successful implementation of content into profession-centric and interprofessional curricula across health disciplines, and therefore was chosen as a model for our work. It helped to inform the development of the shared sex and gender health education tenets described in this article.

In its *Core Competencies for Interprofessional Collaborative Practice: 2016 Update*, the IPEC reiterated eight reasons why using an interprofessional approach for developing core competencies is so important. Most of these reasons are equally applicable to, and act as a driving force for, the development of shared sex and gender health education tenets. Our interprofessional group was especially committed to two of the reasons stated by the IPEC.

- Reason 1: “Create a coordinated effort across the health professions to embed essential content in all health professions education curricula,” which is accomplished through the development of consistent essential elements that all health professions could incorporate into their curriculum with a shared interprofessional goal, and
- Reason 2: “Provide the foundation for a learning continuum in interprofessional competency development across the professions and the lifelong learning trajectory,” which is accomplished through the creation of sex and gender health tenets that clinicians in all health professions could use individually as well as to optimize collaboration.

## Tenets for Sex and Gender Health Education Across Health Professions

Following the 2018 summit, a draft of sex and gender specific learning goals was developed and adopted by the LWBIWH and presented at the American Association of Colleges of Pharmacy annual meeting in 2019 by Dr. Rebecca Sleeper (Table 1). These learning goals became the basis for developing draft tenets by facilitated interprofessional discussion groups at the 2020 Sex and Gender Health Education Summit. The revised drafts from each discussion group were reviewed and edited by an interprofessional working group (article co-authors) to produce four overarching tenets, which are principles to guide this work (Table 2). The group was chosen during the summit and represented most of the health care professions, including dentistry, medicine, nursing, occupational therapy, and pharmacy. Convenings took place, virtually and through multiple email correspondences, until consensus was reached by the group on the recommended tenets.

**Table 1.** Knowledge and Skills for Sex and Gender Specific Health Education

What all health professionals should know how to do
Define accepted SGSH terminology
Differentiate male and female anatomy/physiology
Identify relevant SGSH epidemiology
Identify sex or gender differences in pathophysiology/clinical presentation
Identify sex or gender differences in therapeutic response
Recognize sex or gender based disparities in access to care in health policy
What all health professionals should be able to do
Search and evaluate SGSH Information
Apply SGSH considerations in clinical decision making and patient care
Incorporate SGSH in scientific inquiry and research design
Teach SGSH information to others (peer health professionals or patients)

SGSH, sex and gender specific health.

**Table 2.** Tenets for Sex and Gender Specific Education of Health Professionals

**1. Demonstrate knowledge of sex and gender health:** Understand and be able to describe terminology, definitions, concepts, and sex and gender differences in anatomy, physiology, and pathophysiology, as well as psycho-socio-cultural factors, behaviors, health systems, and social determinants of health
**2. Evaluate literature and the conduct of research for incorporation of sex and gender:** Critically evaluate literature, including guidelines, to identify sex and/or gender disaggregation and analysis of data, appropriateness of conclusions, and identification of gaps in knowledge. When conducting research, include both males/men and females/women and disaggregate, analyze, and report data by sex and/or gender as appropriate
**3. Incorporate sex and gender considerations into decision making:** Apply sex and gender health specific epidemiology, pathophysiology, clinical presentation, therapeutic responses, and health care-seeking behavior to clinical decision making, and care
**4. Demonstrate patient advocacy with respect to sex and gender:** Promote respect for all persons by ensuring that individual sex and gender variables are incorporated into interpersonal interactions and the approach to care, recognizing the intersectionality of these variables with race, sexual orientation, socioeconomic demographics, employment, and other social determinants of health. Working collaboratively with all health professions to deliver individualized sex and gender specific care through a shared interprofessional model is ideal

These tenets provide guidance for establishing the basis for an SGSH education curricula, which can readily be incorporated into interprofessional educational activities as well as within individual professions. Academic leaders and institutions may use these tenets to represent the knowledge, skills, and abilities that should be acquired by all students and trainees in the health care professions before graduation.

**Tenet 1** *Demonstrate knowledge of sex and gender health*. This tenet requires learners to understand basic definitions about SABV and gender as a sociocultural variable to inform how to deliver personalized care to men, women, and sex and gender minority persons. Sex and gender differences in physiology and pathophysiology should be understood at a fundamental level in each body system.
**Tenet 2** *Evaluate literature and the conduct of research for incorporation of sex and gender*. This tenet focuses on research and the need to critically appraise the research literature to assess whether studies include both males/men and females/women as appropriate, and that the data presented have been disaggregated and analyzed by sex and gender. Graduates of health sciences programs should understand the limitations of research that does not include sex and/or gender data and the impact of these limitations on translating research results into patient care. Sex and gender concepts must also be included whenever possible in the methodology and design of learners' future research studies.
**Tenet 3** *Incorporate sex and gender considerations into decision making*. The foundational principles of SGSH knowledge should be applied in all aspects of therapeutics and clinical decision making. Developing a sex and gender lens as a cognitive framework will help mitigate both conscious and unconscious biases, improve patient care, reduce medical errors, and decrease healthcare costs associated with inappropriate testing and treatment.
**Tenet 4** *Demonstrate patient advocacy with respect to sex and gender*. This tenet charges students and healthcare professionals to be advocates for their patients by ensuring that individual sex and gender factors are incorporated into interpersonal interactions. Working collaboratively with all health professions to deliver individualized sex and gender specific care through a shared interprofessional model is ideal. Students should also understand the intersectionality of these variables with race, ethnicity, sexual orientation, socioeconomic demographics, employment, and immigration status, as well as other social determinants of health. Most profession-specific competencies include topics on professionalism such as respect for persons and the general welfare of their patients. This last tenet makes the point that these competencies should also incorporate sex and gender considerations.

### Advocacy beyond educational tenets: a call to action

Advocating for the inclusion of SABV and gender as a sociocultural variable into health professions at all levels is a critically important component of SGSH. While the above tenets are focused on educators and learners, it is important to align the curricular content with educational principles that can be taught and measured. We encourage students, educators, and other health professionals at all levels to promote the inclusion of SGSH—within their schools, communities, local, state, and national policies, and beyond. Doing so through an interprofessional approach will strengthen the efforts and improve success in implementation. Advocacy beyond the educational tenets will be an essential tool to ensure that these tenets reach educators and learners of all health disciplines.

## Discussion: Implementation of Sex and Gender Specific Tenets

Three significant barriers limit universal adoption and integration of sex and gender concepts into education curricula across the health professions: (1) disparate levels of awareness and understanding of what SGSH education means, both inside and outside the academic community, (2) variability in academic competencies and accreditation requirements for training programs among the different health professions, and (3) lack of consistent disaggregation of research data based on sex and/or gender to inform education initiatives.

To overcome these barriers, it is important that champions of this work speak with a clear, concise message in advocating for the necessity of applying a sex and gender lens in all medical and health professions education.^9^ Doing so through interprofessional collaborations and networks strengthens and amplifies a unified message by assuring it moves beyond the scope of an individual profession and allows focused coordinated attention on how SGSH improves patients' health. It also increases precision in concepts and recommendations as insights from different professions require negotiation of meanings and goals among group members. The consensus tenets presented provide a unified message that demonstrates the meaning of SGSH education.

The next step would be to use the tenets to develop operationally specific competencies or competency statements in sex- and gender-based health aligned to each health profession, benchmarks, or other measurement tools for assessment of learners. Interprofessional collaborations will facilitate dissemination of profession-specific best practice recommendations and competencies.^23,24^ Adapting curricula to integrate sex and gender specific competencies ensures that students, trainees, and researchers learn to consider sex and gender aspects of health and disease in their professional careers. Furthermore, delivering the SGSH curriculum in an interprofessional format with common language is ideal. A subsequent step will be for organizations to design interprofessional educational activities in which similar SGSH competencies across professions will enable interprofessional team-based learning.

To facilitate widespread implementation of sex and gender health tenets, existing resources highlighted during the 2020 SGHE Summit such as the NIH ORWH e-learning courses, the U.S. Food and Drug Administration Office of Women's Health webinars, the LWBIWH SGSH curriculum, and the Texas Tech University Health Sciences Center School of Pharmacy's Interprofessional Mini-Series are helpful. A useful medical textbook, *How Sex and Gender Impact Clinical Practice: An Evidence-Based Guide to Patient Care*, contains a six-step model for teaching medical students how to care for patients using a sex and gender approach.^25^ In addition, the AMWA has compiled a comprehensive online collection of sex- and gender-focused resources through its Sex and Gender Health Collaborative.

While most of the available resources come from medicine, they can be adapted and used interprofessionally.^26^ Importantly, current educational materials must be updated as new evidence of sex and gender differences emerges from research studies. A timely example comes from the 2020 COVID-19 pandemic, with data showing lower mortality among women, possibly related to sex-based hormonal and/or immune-mediated advantages in women with regard to protection from the SARS-CoV-2 virus, as well as sociocultural differences in risk preferences, gendered roles, and behaviors.^27,28^

Also important is full adoption and implementation of the Sex and Gender Equity in Research guidelines or similar tools by editors and reviewers of journals and textbooks.^29^ These guidelines provide a framework for research that considers sex and gender, as well as for evaluation of the extent to which sex and gender have been incorporated into submitted articles. Assessment of the use of sex and gender principles in research and research reports must be universally applied and used as part of the article evaluation process. Reporting on the sex and gender implications of research results must be accepted as standard practice by experts and by print and online media.

## Conclusion

SABV has an impact on anatomy and physiology, symptoms, diagnosis, pharmacokinetic and pharmacodynamic responses to treatment, and health outcomes. Gender as a sociocultural variable impacts risk factors for disease—for example nutrition, physical activity, alcohol use, perception of disease by the patient and the health care practitioner, and willingness and ability of an individual to seek health care and follow prescribed management and health behaviors. Gender also influences biology through epigenetic processes. Intersecting with sex and gender are other factors such as race, ethnicity, sexual orientation, socioeconomic factors, and employment, as well as social determinants of health. Currently, SGSH concepts are not routinely incorporated into health education or clinical practice. Systematic education about sex and gender across health disciplines is critical to universal adoption in clinical practice.

To facilitate sex and gender specific education across and among health professions, four tenets are presented for use by students, faculty, and curriculum leaders. The interprofessional collaborative process that produced these tenets is expected to facilitate a uniform voice for integration of sex and gender content into all health professions curricula and enhance and reinforce interprofessional practice. Communication of the tenets through interprofessional networks should strengthen and amplify the message. Such education is expected to advance the health of all individuals, be they women, men, or sex and gender minority persons, which will help close gaps in health disparities.
